# Osteonecrosis of the Jaws Associated with Herpes Zoster Infection: A Systematic Review and a Rare Case Report

**DOI:** 10.3390/microorganisms12081506

**Published:** 2024-07-23

**Authors:** Antonio Mancini, Fabrizio Chirico, Angelo Michele Inchingolo, Fabio Piras, Valeria Colonna, Pierluigi Marotti, Claudio Carone, Alessio Danilo Inchingolo, Francesco Inchingolo, Gianna Dipalma

**Affiliations:** 1 Department of Interdisciplinary Medicine, University of Bari “Aldo Moro”, 70124 Bari, Italy or antonio.mancini@uniba.it (A.M.); or a.inchingolo3@studenti.uniba.it (A.M.I.); or fabio.piras@uniba.it (F.P.); or valeria.colonna@uniba.it (V.C.); or pierluigi.marotti@uniba.it (P.M.); or claudio.carone@uniba.it (C.C.); or a.inchingolo1@studenti.uniba.it (A.D.I.); or gianna.dipalma@uniba.it (G.D.); 2 U.O.C. Maxillofacial Surgery, University of Campania ‘Luigi Vanvitelli’, 81100 Caserta, Italy; fabrizio.chirico@policliniconapoli.it

**Keywords:** osteonecrosis, Herpes Zoster, HSV infection, virus, dentistry

## Abstract

The investigation’s goal was to obtain further knowledge about the connection between *Herpes Zoster* infection and dentistry therapy for the osteonecrosis of the jaws, combining the review with a case report relevant to the purpose. It is important to study this association because it is a possible additional factor to be considered in the causes of the osteonecrosis of the jaws. We limited our search to English-language papers published between 1 January 2004 and 7 June 2024 in PubMed, Scopus, and Web of Science that were relevant to our topic. In the search approach, the Boolean keywords “Herpes Zoster AND osteonecros*” were used. Results: This study analyzed 148 papers from Web of Science, PubMed, and Scopus, resulting in 95 articles after removing duplicates. Of these, 49 were removed because they were off topic, and 46 were confirmed. This study includes a qualitative analysis of the final 12 articles, removing 34 articles that were off topic. The literature highlights severe oral complications from *Herpes Zoster* reactivation, emphasizing the need for early diagnosis, comprehensive management, and multidisciplinary care. Treatment strategies include antiviral therapy, pain management, surgical debridement, and antibiotics. Immunocompromised individuals require vigilant monitoring and balanced immunosuppressive therapy. Further research is needed to enhance therapeutic approaches.

## 1. Introduction

*Herpes simplex virus* (*HSV*) infections are widespread, affecting a significant portion of the global population [1,2,3]. *HSV* is a DNA virus that is mostly divided into two types: *HSV-1*, which usually causes orolabial herpes, and *HSV-2*, which is more frequently the cause of genital herpes [4,5,6]. *HSV* is a member of the *Herpesviridae* family of viruses [7,8,9,10]. While *HSV* is primarily recognized for its mucocutaneous presentations, there has been a growing interest in recent years regarding its possible involvement in systemic disorders, such as osteonecrosis [11,12,13,14,15]. This distinction is important because, although they belong to the same family, the viruses generate diverse diseases and clinical manifestations. The focus on *HSV* draws attention to its increasing significance beyond conventional mucocutaneous symptoms and calls for more research into viral etiologies in systemic diseases like osteonecrosis.

Avascular necrosis, also known as osteonecrosis, is a medical condition where bone tissue dies from inadequate blood flow [16,17,18,19,20]. If left untreated, this illness can lead to severe functional disability, the collapse of the bones, and joint discomfort [21,22,23,24,25,26].

In the United States alone, the incidence of osteonecrosis is significant, with an estimated annual incidence ranging from 10,000 to 20,000 cases, and it is particularly common in individuals aged 30 to 50.

Osteonecrosis has an incidence rate ranging from approximately 1.4 to 3.0 per 100,000 person-years, with the hip being the most commonly diagnosed site, accounting for about 75.9% of cases. Significant risk factors include corticosteroid use, which triples the risk of osteonecrosis, and other conditions such as osteoporosis, cancer, and connective tissue diseases [27].

While direct associations between *HSV* and osteonecrosis are less frequently documented, a study revealed that viral infections, including those caused by herpesviruses, play a role in the non-traumatic etiology of osteonecrosis. Continued research is necessary to better understand the prevalence of *HSV* in osteonecrosis cases and its impact on bone health [28].

Osteonecrosis has a complicated and multifaceted etiology, with both traumatic and non-traumatic causes playing a role in its development [29,30,31,32]. Viral infections, especially those brought on by herpesviruses, are among the non-traumatic causes that have been linked to the pathophysiology of osteonecrosis [32,33,34,35,36]. Osteonecrosis and *HSV* infection are thought to be related by inflammatory reactions that cause vascular damage, direct viral cytopathic effects on bone cells, and autoimmune reactions that worsen the death of bone tissue [37,38,39,40,41]. Osteonecrosis may persist and progress longer if chronic, repeated episodes of *HSV* are allowed to establish latency and reawaken on a periodic basis [42,43,44,45].

Hospital admissions are significantly impacted by *Herpes Zoster (HZ)*, often known as shingles. Research indicates that it may result in a significant number of hospital admissions, particularly in elderly and immunocompromised people. Hospitalizations associated to *HZ* have been found to rise with age, which is consistent with the virus’s increased prevalence in the elderly [46].

An Italian study, for example, found that *HZ* places a significant strain on hospital resources, with hospitalization rates among people 50 years of age and older being particularly high. This pattern highlights the high expense of healthcare and the necessity of efficient administration and preventative measures. Although the varicella vaccine has changed the epidemiology of *HZ* in the US, hospital admission rates have lately plateaued or even decreased, possibly as a result of increased vaccination rates and early therapeutic interventions [47].

The aforementioned data emphasize the vital necessity of continuous surveillance and public health campaigns to effectively control and alleviate the effects of *HZ* on healthcare systems across the globe.

The studies analyzed in this research underscore diverse *RHS* symptoms and stress early diagnosis, comprehensive care, and interdisciplinary treatment. Tailored therapies like surgery and antivirals are crucial for managing oral issues. Kaur, Cloarec, and Song addressed *VZV* challenges in *HIV* patients, advocating for vigilant monitoring, prompt intervention, and multidisciplinary care [48,49,50].

Several publications highlight significant oral complications from *VZV* reactivation in immunocompetent patients, emphasizing early identification and multidisciplinary management. Successful outcomes in *VZV*-related osteonecrosis hinge on surgical intervention, antibiotics, and tailored treatments, necessitating ongoing research and innovative approaches [17,18,51].

In this systematic analysis, we also describe a thorough case report: a unique instance of osteonecrosis that may have been caused by an infection with the *herpes simplex virus*. By outlining this patient’s clinical presentation, diagnostic process, and case management, we wish to raise awareness of the possible connection between *HSV* and osteonecrosis. This study emphasizes how crucial it is to take viral etiologies into account when treating individuals who have osteonecrosis that cannot be explained and recommends more investigation to determine how *HSV* functions in the pathophysiology of this crippling bone disease.

Our goals with this review and case report are to encourage medical professionals to perform comprehensive diagnostic evaluations for patients who present with osteonecrosis, especially if they have a history of recurrent herpesvirus infections, and to add to the growing body of research on the systemic effects of these infections.

## 2. Materials and Methods

### 2.1. Protocol and Registration

This review was carried out in accordance with PRISMA (Preferred Reporting Items for Systematic Reviews and Meta-Analyses) guidelines, and it was registered under the number CRD564003 on PROSPERO (The International Prospective Register of Systematic Reviews) [52]. In addition to the review, a case report of a patient with *HZ* associated with the clinical manifestation of osteonecrosis at the mandibular level was incorporated into the article.

### 2.2. Search Processing

We limited our search to English-language papers published between 1 January 2004 and 7 June 2024 in PubMed, Scopus, and Web of Science that were relevant to our topic. In the search approach, the Boolean keywords “*Herpes Zoster* AND osteonecros*” were used. We selected these phrases because they most accurately reflected our investigation’s aim, which was to gain additional insight into the interaction between *HZ* infection and dentistry therapy for the osteonecrosis of the jaws (Table 1).

### 2.3. Inclusion Criteria

Three reviewers evaluated all relevant papers based on the following chosen criteria: (1) solely human subjects in studies; (2) complete text; and (3) scientific studies evaluating the management of the removal of the osteonecrosis of the jaws. The following process was used to construct the PICO model:

- Criteria: Application in the present study;
- Population: Human subjects with the osteonecrosis of the jaws with *HZ* infection;
- Intervention: Dentistry treatment for the removal of osteonecrosis;
- Comparison: Control group;
- Outcome: Evaluation of healing;
- Study design: Case reports.

### 2.4. Exclusion Criteria

Articles written in languages other than English, ineligible study designs, ineligible outcome measures, ineligible populations, case studies, reviews, and animal studies were among the exclusion criteria.

### 2.5. Data Processing

Two reviewers (F.P. and V.C.) searched the database to extrapolate the studies and assessed their quality independently, according to selection criteria. During the screening phase, we excluded articles that did not fit the topic by reading the manuscript title and the abstract. The full texts of the remaining articles were read to conduct an eligibility analysis, according to the inclusion criteria. The selected articles were downloaded in Zotero (version 6.0.15). Any discrepancies between the two authors were resolved by consulting a senior reviewer (F.I.).

### 2.6. Article Identification Procedure

The appropriateness evaluation was performed independently by two reviewers, F.I. and F.P. An additional manual search was conducted to increase the number of articles available for full-text analysis. English-language articles that met the inclusion criteria were taken into consideration, and duplicates and items that did not qualify were marked with the reason that they were not included.

### 2.7. Study Evaluation

The article data were independently evaluated by the reviewers using a special electronic form designed according to the following categories: authors, year of study, aim of the study, materials and methods, and results.

### 2.8. Quality Assessment

Two reviewers, F.P. and F.I., evaluated the included papers’ quality using the ROBINS-I tool (Cochrane Bias Methods Group and the Cochrane Non-Randomised Studies of Interventions Methods Group—Creative Commons Attribution-NonCommercial-NoDerivatives 4.0 International License). In order to evaluate the possibility of bias in the outcomes of non-randomized trials comparing the health impacts of two or more therapies, ROBINS-I was created. Each of the seven evaluated points was given a bias degree. F.I., the third reviewer, was consulted in the case of disagreement until a consensus was reached. The reviewers were instructed on how to use the ROBINS-I tool and adhered to the guidelines in order to assess the potential for bias in seven different domains: confounding, participant selection, intervention classification, deviations from intended interventions, missing data, outcome measurement, and choice of reported results. Discussion and consensus were used to settle any differences or conflicts amongst reviewers in order to improve the assessments’ objectivity and uniformity. In situations when agreement could not be reached, the final decision was made by a third reviewer. An extensive assessment of potential biases in the non-randomized studies included in this study was made possible by the use of ROBINS-E for bias assessment. This contributed to the overall evaluation of the caliber and dependability of the results by pointing out the evidence base’s advantages and disadvantages. The writers of this review were able to reach more informed interpretations and conclusions based on the facts at hand by taking the risk of bias into account.

## 3. Results

**Figure 1:**
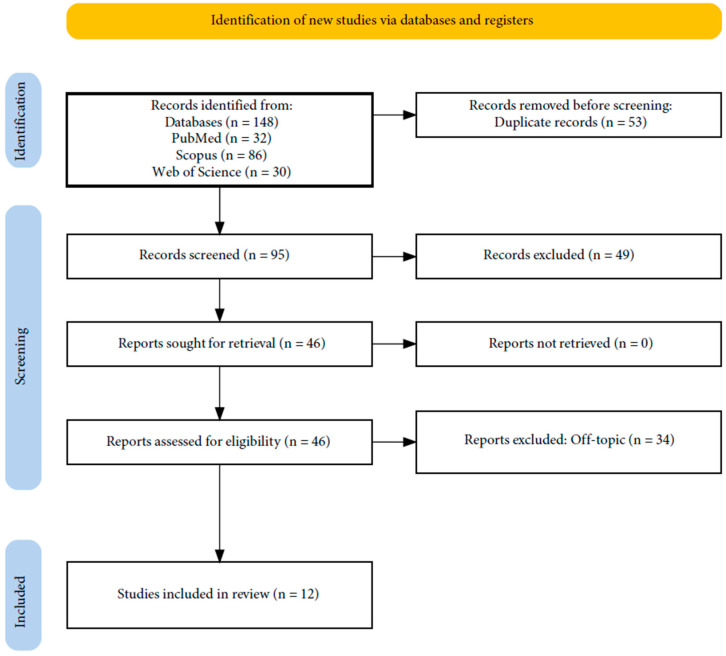
PRISMA flowchart of the literature search and article inclusion process.

A total of 148 papers were obtained from the databases Web of Science (30), PubMed (32), and Scopus (86). This resulted in 95 articles after eliminating duplicates (53). Next, 49 entries were eliminated after their titles and abstracts were examined because they did not fit the inclusion criteria. The writers were able to successfully obtain the remaining 46 papers and confirm their eligibility; 34 items were eliminated as a result of this process because they were off topic. The qualitative analysis of the 12 final articles is included in this study (Figure 1). Each study’s findings are presented in Table 2.

### Quality Assessment and the Risk of Bias in the Included Articles

**Figure 2:**
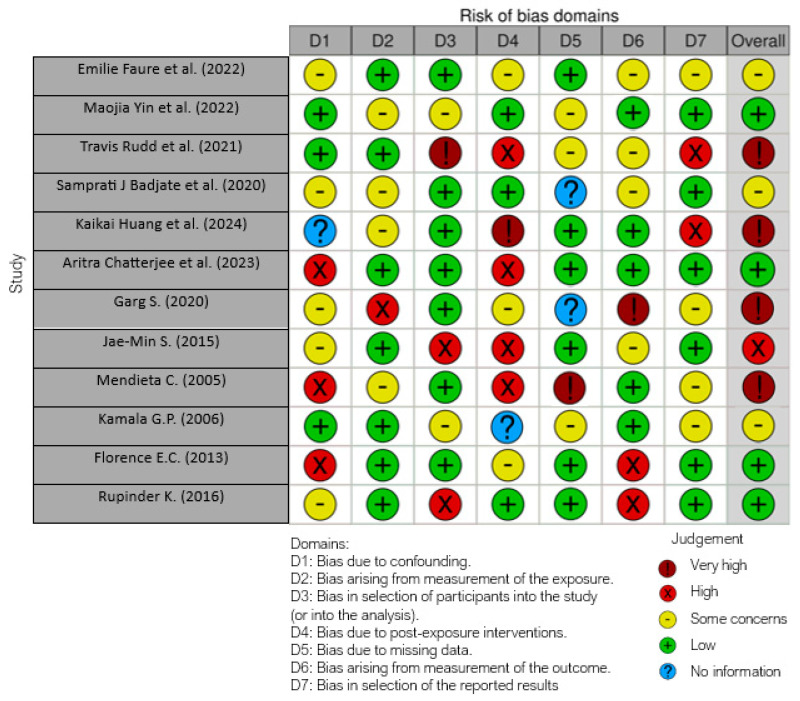
Bias assessment by Robins tool [14,17,18,48,50,51,54,55,56,57,58,59].

The risk of bias in the included studies is reported in Figure 2. Regarding the bias due to confounding, most studies had some concerns. The bias arising from measurement is a parameter with low risk of bias. Many studies had low risk of bias due to bias in selection of participants. Bias due to post exposure could not be calculated due to high heterogeneity. The bias due to missing data was low in many studies. Bias arising from measurement of the outcome was low. Bias in the selection of the reported results was low in most studies. The final results show that one study had a high risk of bias, four had a very high risk of bias, and four had a low risk of bias.

## 4. Discussion

*HZ*, commonly known as shingles, is caused by the reactivation of the *varicella zoster virus* (*VZV*), which lies dormant in the dorsal root ganglia after an initial chickenpox infection [60,61,62,63,64]. This viral reactivation can lead to various complications, particularly when the trigeminal nerve is involved, potentially resulting in severe oral health issues [65,66,67,68,69]. Osteonecrosis, or the death of bone tissue, is a particularly concerning complication of *HZ*, often manifesting in the maxillofacial region and leading to significant morbidity [70,71,72]. This paper explores the incidence and management of *HZ*–related osteonecrosis across different patient populations, including those with Ramsay Hunt Syndrome (RHS), AIDS, and generally healthy individuals [73,74,75,76].

### 4.1. Patients with Ramsay Hunt Syndrome

Ramsay Hunt Syndrome (RHS) is a severe neurological condition resulting from the reactivation of the *VZV* within the trigeminal nerve ganglia [77,78,79,80,81]. It affects adults, particularly those over 60 (about 5 out of every 100,000 people each year), and is primarily caused by impaired immune systems and stress. The incidence is significant, with a higher rate among the elderly population. This reactivation can lead to a range of complications, including osteonecrosis and tooth exfoliation. The cases presented by Garima Singh et al., Maojia Yin et al., Travis Rudd et al., and Samprati J. Badjate et al. provide insight into the diverse manifestations and management strategies for RHS [82,83,84,85]. Garima Singh et al. reported a case involving a 13-year-old girl who developed alveolar osteonecrosis and subsequent tooth loss following *VZV* infection [56,86,87]. This case highlights the significant risk of these complications even in younger patients and underscores the critical need for early diagnosis and comprehensive management. The young age of the patient emphasizes the importance of maintaining a high index of suspicion for *VZV*-related oral complications across all age groups [88,89]. In contrast, Maojia Yin et al. described a 50-year-old man with RHS characterized by severe facial and mandibular pain, rapid tooth loss, and mild facial paralysis [18,90,91]. The treatment involved a tailored regimen including gabapentin, pregabalin, paracetamol–tramadol, and eperisone hydrochloride, supplemented by periodic debridement of necrotic bone and prophylactic antibiotics [92,93,94,95]. This multidisciplinary approach was crucial in managing pain and controlling the progression of osteonecrosis, highlighting the necessity of individualized treatment strategies for RHS patients [96,97,98,99,100]. Travis Rudd et al. documented a case of a 59-year-old man with granulomatosis with polyangiitis who developed severe mandibular osteonecrosis and RHS subsequent to *VZV* reactivation [59,101,102]. Despite aggressive treatment with intravenous antivirals and surgical debridement, the patient experienced persistent symptoms, underscoring the chronic nature of RHS-associated complications [103,104,105,106]. This case highlights the importance of vigilant long-term follow-up and continuous interdisciplinary care in managing complex RHS scenarios effectively [107,108,109,110]. Samprati J. Badjate et al. presented an 86-year-old male with RHS, post-herpetic neuralgia, and bilateral osteonecrosis involving both the maxilla and mandible [17,111]. The treatment comprised initial antiviral therapy followed by surgical debridement and antibiotic administration, resulting in favorable wound-healing outcomes [112,113,114]. This case emphasizes the pivotal role of timely intervention and a collaborative medical approach in mitigating severe *VZV*-related complications in the craniofacial region [115,116,117]. In summary, the cases presented by Garima Singh et al., Maojia Yin et al., Travis Rudd et al., and Samprati J. Badjate et al. [17,18,53,56] highlight the diverse manifestations of RHS and the importance of early diagnosis, comprehensive management, and multidisciplinary care. The varying ages of the patients and the severity of their symptoms underscore the need for tailored treatment strategies and vigilant long-term follow-up [118,119]. These cases also emphasize the critical role of antiviral therapy, surgical debridement, and antibiotic administration in managing the oral and maxillofacial complications of RHS.

### 4.2. Patients with AIDS

Individuals with AIDS exhibit a significantly elevated incidence of *VZV* reactivation due to immunosuppression [120,121]. Rupinder Kaur emphasized that, while *VZV* prevalence in the general population is 5.4%, it can be up to 10 times higher in *HIV*-positive individuals [48,122,123,124,125]. This heightened susceptibility underscores the critical need for vigilant monitoring and prompt intervention in this vulnerable patient cohort [126,127,128,129]. Nicolas Cloarec discussed the administration of azathioprine in *HIV*-infected patients under highly active antiretroviral therapy (HAART), noting its safe usage with close immunological monitoring [49]. However, he cautioned about potential adverse effects, including complications from *VZV* reactivation, emphasizing the delicate balance required in managing immunosuppressive therapies in *HIV*/AIDS patients [130,131,132]. Furthermore, Jae-Min Song’s literature review highlighted severe oral manifestations of *VZV* infection in immunocompromised patients, such as those with *HIV*/AIDS [50]. Involvement of the trigeminal nerve branches can lead to complications such as acute pulpitis, dental pain, root resorption, and periapical lesions, necessitating immediate antiviral treatment and effective pain management strategies to mitigate the risk of post-herpetic neuralgia and further oral deterioration [133,134,135]. In summary, the discussions by Rupinder Kaur, Nicolas Cloarec, and Jae-Min Song highlight the unique challenges posed by *VZV* reactivation in *HIV*-positive individuals. The elevated incidence of *VZV* infection in this population necessitates vigilant monitoring and prompt intervention [83,106,136,137,138]. The use of immunosuppressive therapies, such as azathioprine, requires careful management to balance the benefits and risks, including the potential for *VZV* reactivation. Additionally, the severe oral manifestations of *VZV* infection in immunocompromised patients underscore the need for immediate antiviral treatment and effective pain management strategies to prevent further complications. These findings emphasize the importance of a multidisciplinary approach in managing *VZV*-related complications in *HIV*/AIDS patients [139,140,141].

### 4.3. Healthy Patients or Those without Either Pathology

Even in immunocompetent individuals, *VZV* reactivation can result in significant oral complications, albeit less frequently and severely compared to immunocompromised patients [142,143,144,145]. Garima Singh et al. noted that, while *VZV* primarily affects older adults, cases like that of the young girl underscore the importance of maintaining a high index of suspicion for *VZV*-related oral complications across all age groups [146,147,148,149]. C. Mendieta’s research further explored the prevalence of *VZV* in the general population and its potential to cause neuralgia and oral lesions [57,150,151,152,153]. This study highlighted that trigeminal nerve involvement can lead to substantial oral health issues such as tooth loss due to alveolar bone necrosis, emphasizing the need for early diagnosis and comprehensive management strategies in healthy individuals [154,155,156,157]. Emilie Faure et al. described an 87-year-old man with uncontrolled diabetes who developed mandibular osteonecrosis following *VZV* infection [51]. Despite his compromised health status, the patient’s condition was successfully managed through surgical resection of necrotic bone and antibiotic therapy, underscoring the importance of addressing underlying health conditions to optimize recovery [110,158,159,160]. Kaikai Huang et al. discussed rare but severe complications of alveolar osteonecrosis associated with *VZV*, stressing the critical need for awareness and prompt treatment to mitigate oral and maxillofacial complications [54]. This review emphasized the necessity for further research into pathogenesis and treatment strategies for *VZV*-related osteonecrosis [161,162,163,164]. Aritra Chatterjee et al. presented a unique case of the osteonecrosis of the jaw (ONJ) following *VZV* infection in a 51-year-old man [55,165,166]. Despite initial symptomatic treatment, the patient developed spontaneous tooth exfoliation and mandibular bone necrosis, necessitating surgical intervention and antibiotic therapy [167,168,169]. This case underscores the complexity of managing postherpetic ONJ and the importance of tailored treatment approaches. Kamala G. Pillai highlighted that *VZV* can affect cranial nerves, particularly the trigeminal nerve, potentially leading to osteomyelitis and spontaneous tooth loss [14,170,171,172]. Her review of cases illustrated rapid disease progression and severe bone destruction, emphasizing the need for preventive measures and early intervention to mitigate these risks [49,173,174,175]. In summary, the discussions by Garima Singh et al., C. Mendieta, Emilie Faure et al., Kaikai Huang et al., Aritra Chatterjee et al., and Kamala G. Pillai highlight the significant oral complications that can arise from *VZV* reactivation in immunocompetent individuals [76,176,177,178]. These complications, ranging from alveolar bone necrosis to osteomyelitis and tooth loss, underscore the importance of early diagnosis and comprehensive management strategies [179,180,181,182]. The successful management of these cases through surgical intervention, antibiotic therapy, and tailored treatment approaches emphasizes the need for a multidisciplinary approach [183,184,185,186]. Additionally, the review by Kaikai Huang et al. highlights the necessity for further research into the pathogenesis and treatment strategies for *VZV*-related osteonecrosis, emphasizing the ongoing need for vigilance and innovative therapeutic approaches in managing these complex conditions [187,188,189,190,191]. In conclusion, the discussed literature underscores the critical importance of early diagnosis, comprehensive treatment, and multidisciplinary care in managing *VZV*-related complications across diverse patient populations [192,193,194,195]. Whether in the context of RHS, *HIV*/AIDS, or healthy individuals, understanding the distinct challenges and tailoring treatment accordingly are crucial for optimizing patient outcomes [196,197,198,199,200,201].

### 4.4. Analysis of Lack of Vaccine

There are a number of reasons why patients with problems from *HZ* may not have had a vaccine, including the following:

1. Access and Availability: Depending on factors like cost, availability, or healthcare infrastructure, certain areas may not have easy access to vaccinations;
2. Vaccine hesitation: Undervaccination can result from vaccine hesitation caused by false information, cultural values, or personal convictions;
3. Immunocompetence: Certain people may not be able to receive vaccinations or may respond less well to them, especially if they have an immunocompromising medical problem;
4. Age-Related Factors: Adults above a particular age should receive the shingles vaccine; however, undervaccination in that age group might occur from a lack of knowledge or availability.

The *VZV* vaccination has the potential to significantly lower the frequency and severity of *HZ* and its associated consequences, such as osteonecrosis [202,203,204]. The possible effects of vaccination are highlighted by the following points:

1. Prevention of Initial Infection

Childhood vaccination against varicella, or chickenpox, helps avoid the virus’s first infection, lowering the possibility that it will lie dormant and reactivate as *HZ* later in life. Immunocompetent people should pay special attention to this as it can drastically lower the general prevalence of *HZ* in the community;

2. Reduction of *HZ* Incidence in Adults

By increasing the immune response to *VZV*, the shingles vaccine, which is advised for individuals over a specific age, can lower the risk of *HZ*. Due to the age-related reduction in immune function, older persons are particularly benefiting from this, since they are more likely to acquire *HZ* and its associated consequences;

3. Mitigation of Complications in Immunocompromised Individuals

Immunocompromised people, such as those with AIDS, may benefit less from vaccination, although it can still provide some protection against *VZV* reactivation and its consequences. In these situations, it is crucial to carefully weigh the patient’s immunological condition as well as the possible advantages and disadvantages of vaccination;

4. Public Health Implications

Reducing the burden of problems connected to *HZ* can be achieved in part by addressing vaccine reluctance, expanding immunization rates through public health initiatives, and enhancing vaccine accessibility. Part of this involves spreading knowledge about the existence of the shingles and chickenpox vaccinations as well as their significance in avoiding *HZ* and its severe symptoms [205,206,207,208,209].

To sum up, immunization against *VZV* is an essential part of the plan to lower the frequency and severity of *HZ* and its side effects, such as osteonecrosis. Public health authorities and healthcare personnel may greatly enhance patient outcomes for a wide range of groups by advocating for children and adult vaccinations.

### 4.5. Study Limitations

It is important to recognize the following limitations while studying and analyzing *HZ* and its associated consequences, especially osteonecrosis, in a variety of patient groups, including those with AIDS, RHS, and normally healthy people:

1. Sample Size and Representativeness: The cases that are being reviewed are drawn from short case series and individual patient reports. This restricts the findings’ applicability to a larger population. To completely comprehend the incidence and prevalence of *HZ*-related osteonecrosis across various patient categories, larger, more representative investigations are required;
2. Selection Bias: Not all patients with *HZ* or RHS may be represented by the instances that are provided. The perception of the normal presentation and severity of problems connected to *HZ* may be skewed to a potential bias towards reporting instances that are more severe or uncommon;
3. Absence of Longitudinal Data: Long-term follow-up data are lacking in many of the examples that are described. Because of this, evaluating the long-term effects and efficacy of different treatment approaches over time becomes difficult. To fully comprehend the chronic nature of issues connected to *HZ* and the long-term effects of therapy, longitudinal studies are required;
4. Variability in Treatment Options: As a reflection of the customized character of care, the treatment options presented differ greatly between instances. Nevertheless, it is challenging to obtain firm judgments regarding the best course of action in terms of therapy. This issue may be addressed with the use of comparative research and standardized treatment methods;
5. Variability in Treatment Choices: The treatment choices offered vary significantly between cases, reflecting the individualized nature of therapy. However, it might be difficult to obtain definitive opinions about the optimal therapeutic approach. Standardized treatment techniques and comparative studies may be used to solve this problem;
6. Immunocompetence Variability: Patients’ immune systems can differ significantly, even within those in the same diagnostic group (AIDS and RHS, as examples). The manifestation and seriousness of issues linked to *HZ* can be influenced by this variability. Research that takes into consideration and evaluates the effects of varying degrees of immunocompetence is required;
7. Geographic and Demographic Variability: It is possible that the examples examined do not accurately reflect populations throughout the world. The frequency, manifestation, and consequences of *HZ* can be influenced by demographic and geographic variables. More extensive global research may yield more thorough understanding;
8. Emerging Therapies and Research: As novel treatments and interventions are being investigated, the area of *HZ* and *VZV*-related problems is changing quickly. The necessity for ongoing updates to knowledge and treatment standards is highlighted by the possibility that the current conversation may not completely capture the most recent breakthroughs.

### 4.6. Clinical Implications

1. Early Diagnosis and Management

The instances discussed highlight how crucial it is to identify issues linked to *HZ* early on and to handle them comprehensively. Across all age ranges and patient demographics, healthcare personnel should keep a high index of suspicion for oral problems connected to *VZV*. For optimal therapy, multidisciplinary care, including oral surgeons, dentists, infectious disease specialists, and pain management experts, is essential;

2. Tailored Treatment Strategies

Personalized treatment regimens are required because of the wide range in severity and manifestation of problems linked to *HZ*. These include the application of antibiotics, surgical debridement, antiviral treatment, and pain control techniques. Vigilant observation and timely action are crucial for patients with impaired immune systems, including those suffering from AIDS, in order to reduce the likelihood of serious consequences;

3. Vaccination

The study emphasizes how crucial it is to get vaccinated against *VZV*. Both the adult shingles vaccine and the childhood chickenpox immunization can dramatically lower the frequency and seriousness of problems connected to *HPZ*. The main goals of public health initiatives should be to combat vaccine hesitancy, improve vaccine accessibility, and raise immunization rates via education;

4. Long-Term Follow-Up

Because certain *HZ*-related consequences are chronic in nature, especially in complicated cases like RHS, ongoing multidisciplinary treatment and long-term follow-up are required to manage enduring symptoms and avert further problems.

### 4.7. Future Prospects

1. Research on Pathogenesis and Treatment

More investigation is required to improve our understanding of the pathophysiology of osteonecrosis linked to *VZV* and to create more potent treatment plans. This involves looking into the processes that lead to nerve involvement and bone loss. To determine standardized treatment procedures and assess the effectiveness of various treatment modalities, clinical trials and comparison studies are necessary;

2. Innovative Therapeutic Approaches

Potential approaches to treating *HZ*-related problems include the creation of novel therapeutic agents and interventions, such as tailored antiviral treatments and regenerative medicine methods. Investigating the possibilities of stem cell therapy and tissue engineering as treatments for osteonecrosis may yield novel approaches;

3. Vaccine Development

It is critical to continue ongoing research on vaccine development, particularly studies on the safety and effectiveness of vaccinations in immunocompromised populations. Investigating the possibilities of novel vaccine formulations and delivery systems is part of this;

4. Global Health Perspectives

More extensive worldwide research is required to comprehend the occurrence and manifestation of *HZ*-related complications worldwide. This will support the creation of thorough global health policies and standards;

5. Technological Advancements

The early diagnosis and monitoring of problems connected to *HZ* may be enhanced by the use of sophisticated diagnostic methods, such as imaging technology and biomarkers. Digital health platforms and telemedicine might improve access to care and help with the better management of long-term illnesses like RHS [210,211].

The reviewed literature offers strong proof that *VZV* infection is relevant to the onset of osteonecrosis. The need for early diagnosis, comprehensive management, and multidisciplinary care is highlighted by the numerous presentations and severe complications observed in different patient categories, such as patients with AIDS, RHS, and healthy persons. The prevention of *VZV*-related problems is mostly dependent on vaccination, and continued research is necessary to create more potent vaccines and treatment plans [212,213].

### 4.8. Case Presentation

**Figure 3:**
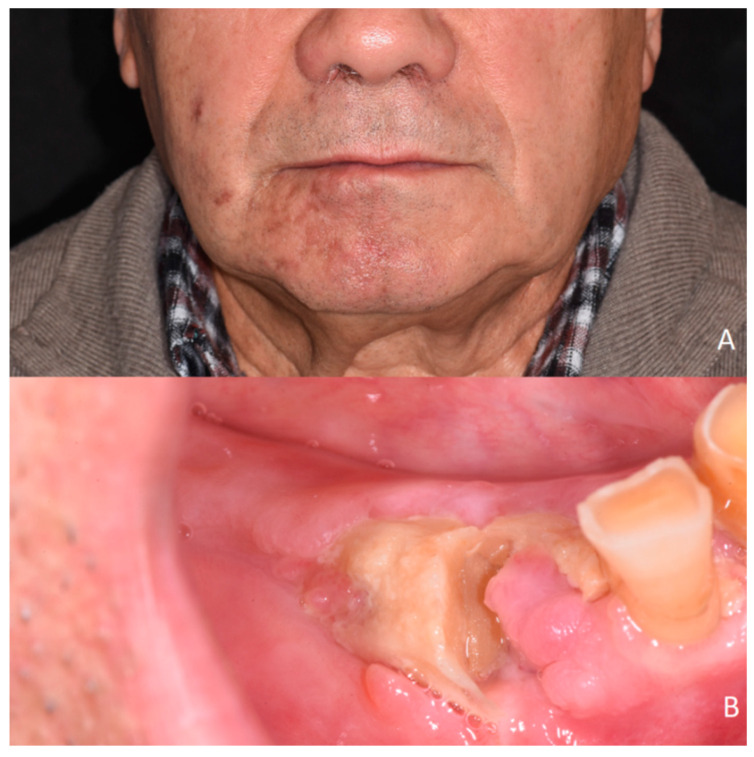
Preoperative clinical view. (**A**) Extraoral view. (**B**) Intraoral view.

A 74-year-old male patient visited the Department of Interdisciplinary Medicine, University of Bari “Aldo Moro”, on 31 January 2018, and his chief complaint was alveolar bone exposure of the right mandible. The patient received dental extractions from the right half of his jaw for destructive caries in the years preceding the onset of osteonecrosis. He presented absence of current pharmacological therapies and absence of current or previous pathologies, both systemic and in the head and neck area. He had an onset of herpetic rashes in the right side of the hemimandibula, concomitant with an area of bone exposure in the right side of the haemimandibula, which persisted after the resolution of skin symptoms (Figure 3A). He had no other relevant medical history. Intraoral examination showed alveolar bone exposure in the #43, #44 area (Figure 3B).

**Figure 4:**
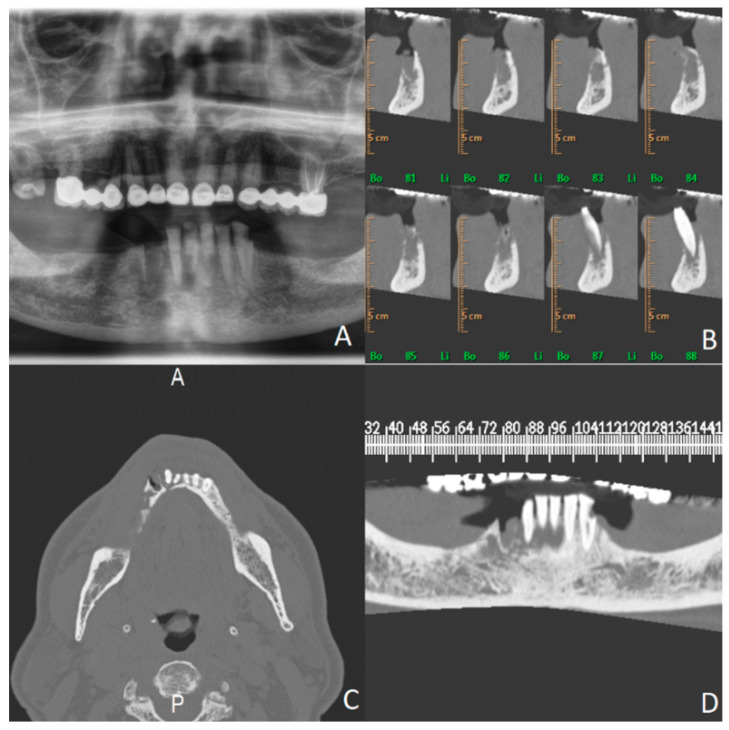
Preoperative radiographs. (**A**) Preoperative panoramic view. (**B**) Pre-operative computed tomography sagittal view. (**C**) Pre-operative computed tomography axial view. (**D**) Pre-operative computed tomography frontal view.

Radiographic examination, according to axial thin-layer scans (1 mm), reconstructed in coronal (panorex) and sagittal oblique planes (cross), showed calcium tone alteration with diffuse rarefaction of the bony trabeculae, with tissue swelling seen on computed tomography (Figure 4).

**Figure 5:**
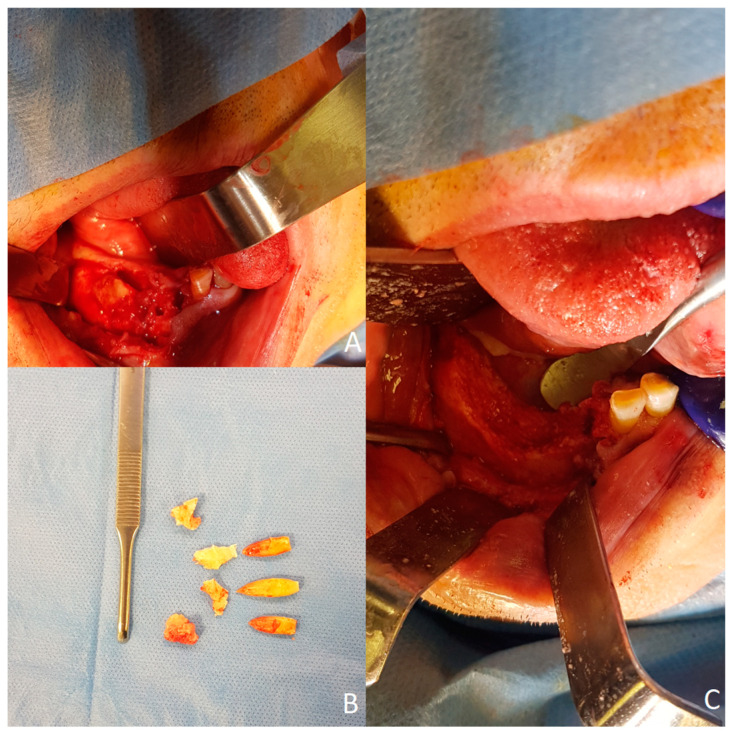
Intraoperative procedures. (**A**) Sequestrectomy and teeth removal were conducted. (**B**) Fragments of necrotic bone. (**C**) A sound bone bed was exposed and primarily closed.

CT findings could be suggestive of ONJ; however, completion of a clinical–anamnestic–instrumental link is required. The patient had no history of receiving a prescription for any medication that affects bone metabolism and had never received radiotherapy. We determined that the inflammation originated from the patients’ teeth; thus, we diagnosed his condition as right mandibular osteonecrosis by trigeminal *HZ*. We treated him with sequestrectomy, and we removed teeth #31-#41-#42 (Figure 5).

**Figure 6:**
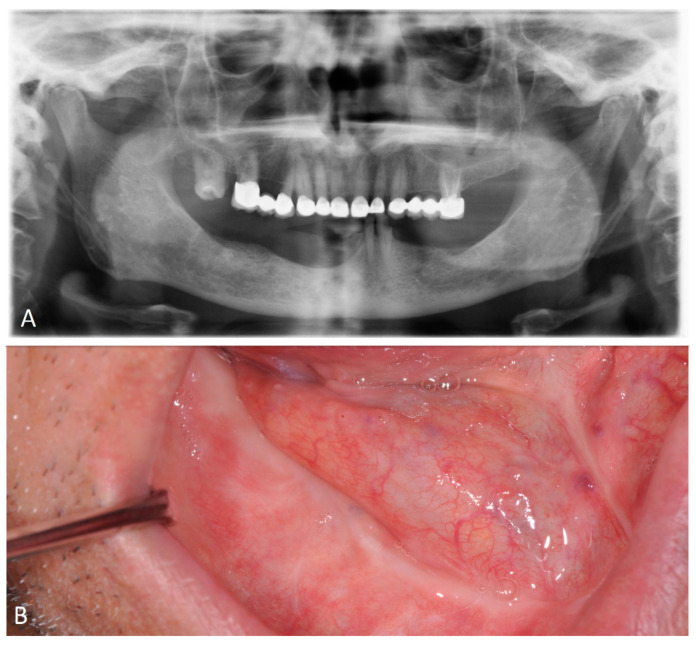
Postoperative examinations. (**A**) Postoperative panoramic view. (**B**) Intraoral view.

After the surgery, a piece consisting of fragments of necrotic bone and inflammatory fibrous tissue was examined. According to histopathology, we observed clumps of necrotic material, fibroconnectival frustules with chronic non-specific inflammatory infiltration, and spicules of bone tissue, partly necrotic, surrounded by fibroconnectival vallo. There was an absence of post-operative complications and any osteogenesis of the area of previous osteonecrosis following resective surgery of the lesion itself. No additional tooth loss, bone exposure, or osteonecrosis occurred during the follow-up period. Also, no other complications, such as postherpetic neuralgia, occurred (Figure 6).

## 5. Conclusions

The reviewed literature and case study highlight the severe oral complications, particularly osteonecrosis, that can arise from *HZ* reactivation, emphasizing the necessity for early diagnosis, comprehensive management, and multidisciplinary care. In Ramsay Hunt Syndrome (RHS) patients, individualized treatment strategies involving antiviral therapy, pain management, surgical debridement, and antibiotics are crucial. Immunocompromised individuals, especially those with AIDS, require vigilant monitoring and a balanced approach to immunosuppressive therapy to prevent severe oral manifestations. Even immunocompetent individuals can experience significant complications, underscoring the need for a high index of suspicion and timely intervention. The 74-year-old male case reinforces the importance of thorough diagnostics and prompt surgical intervention for successful outcomes. Overall, tailored treatment, early diagnosis, and multidisciplinary care are vital for managing the complex complications of *HZ*, with further research needed to enhance therapeutic approaches.

## Figures and Tables

**Table 1:** Indicators for database searches.

Articles screening strategy	KEYWORDS: “A”: Herpes Zoster; “B”: osteonecros*;
	Boolean Indicators: “A” AND “B”
	Timespan: 1 January 2004, to 7 June 2024
	Electronic databases: PubMed; Scopus; Web of Science.

**Table 2:** A descriptive item selection summary.

Authors (Year of Publication)	Type of Study	Aim of the Study	Materials	Results
Emilie Faure et al. (2022) [51]	Case report	To report a rare case of mandibular osteonecrosis following *HZ* infection and review relevant literature.	87-year-old Caucasian man affected by uncontrolled diabetes (*HbA1c*: 8.8%), complicated by microangiopathy and macroangiopathy, myocardial infarction, and integrative omics-metabolic analysis (IOMA) that required a femoral bypass. Reported discomfort in wearing his prosthesis, responsible for feeding difficulties. Intraoral examination revealed a large bone exposure in the previously ulcerated area, measuring 2 cm × 1 cm, and showing necrotic alveolar bone associated with peripheral suppuration.	Osteonecrosis of the right mandibular alveolus was identified; it was treated with antibiotics, extraction of the tooth, and excision of necrotic bone. Intermaxillary fixation and a titanium reconstruction plate were used to manage the following pathological fracture, and a follow-up revealed satisfactory bone healing.
Maojia Yin et al. (2022) [18]	Case report and literature review	To review the literature, report a case of *HZ*, and cause mandibular alveolar bone necrosis.	A 50-year-old man who requested pain medication for facial and ear pain also developed necrosis, blister rash, hearing loss, and facial paralysis.	Trigeminal *HZ* and RHS were identified after tumors and other infectious illnesses were ruled out.
Travis Rudd et al. (2021) [53]	Case report and literature review	Describe a case of mandibular alveolar bone necrosis and Ramsay Hunt Syndrome that occurred after *HZ* and review relevant literature.	*HZ* affecting the mandibular branch of the trigeminal nerve manifested in a 59-year-old male with a medical history that included hypertension, hyperlipidemia, and granulomatosis with polyangiitis (Wegener). He also experienced impaired hearing in his right ear as a result of Ramsay Hunt Syndrome (RHS) and peripheral Bell palsy on the right side. Spontaneous tooth loss and osteonecrosis in the right mandible, which were linked to a severe case of facial *HZ* rash and ongoing RHS symptoms.	The case emphasized the prevalence of mandibular osteonecrosis and Ramsay Hunt Syndrome post-*HZ*; treatment included antiviral and antibiotic medication, leading to clinical improvement.
Samprati J Badjate et al. (2020) [17]	Case report and literature review	Reviewing the relevant literature and reporting an unusual case of Ramsay Hunt Syndrome aggravated by edentulous maxilla and mandible osteonecrosis.	An 86-year-old patient with facial nerve paralysis post HZI, edentulous arches, fibrosis, tongue depapillation, white scarring, blood-encrusted areas, low-grade lower motor neuron facial palsy, and exposed alveolar bone.	The therapy of Ramsay Hunt Syndrome and related osteonecrosis was described in depth in the case report. Significant clinical improvement resulted from the use of antibiotics and antiviral medication during treatment.
Kaikai Huang et al. (2024) [54]	Case report and literature review	To investigate how infection contributes to the development of alveolar osteonecrosis after zoster facial herpes.	A 67-year-old man experienced facial hemorrhage and alveolar osteonecrosis.	The study suggested that infection might significantly contribute to the development of alveolar osteonecrosis post-*HZ*. The case report detailed diagnosis and treatment with antibiotics and antiviral therapy, showing positive outcomes.
Aritra Chatterjee et al. (2023) [55]	Case report	To describe and elaborate on a delayed case of HZI that resulted in mandibular osteonecrosis.	Following three months of HZI, a 51-year-old male patient showed signs of spontaneous exfoliation of several teeth and a subsequent pathological fracture on the right side of the lower jaw.	No signs of recurrence were seen throughout the patient’s year-long follow-up. Osteonecrosis after heart-lung transplantation is a distinct but uncommon presentation that needs to be recognized right away.
Garima S. (2020) [56]	Case report and literature review	To highlight the importance of early diagnosis and appropriate management of the condition, as well as the need for a detailed medical and dental history to facilitate timely intervention.	The subject of the study is a 13-year-old patient who presented with a rare condition associated with HZI. Specifically, the patient experienced alveolar osteonecrosis and tooth exfoliation, which are uncommon oral complications of *HZ*, particularly in pediatric patients.	The patient underwent symptomatic pharmacological therapy for a week, with an improvement in symptoms. A prosthetic rehabilitation was performed with a removable partial denture for tooth 11.
Jae-Min S. (2015) [50]	Case report and literature review	The study aims to highlight the rare but significant dental complications associated with *HZ* infection, particularly in the mandibular branch, and to discuss the management and treatment options.	The subject of the study is a 64-year-old male patient who presented with osteonecrosis with bone exposure in the left mandible as a complication of *HZ* infection involving the mandibular branch of the trigeminal nerve.	The treatment with sequestrectomy and removal of teeth 31–35 was successfully performed, and, during the follow-up period, there were no further episodes of tooth loss, bone exposure, or osteonecrosis, nor complications such as postherpetic neuralgia.
Mendieta C. (2005) [57]	Case report and literature review	To discuss the clinical manifestations, treatment, and potential mechanisms underlying this rare complication of *HZ* infection.	A 63-year-old woman with *HZ* infection involving the trigeminal nerve with advanced alveolar bone loss observed around teeth 27 and 28.	The patient was treated with oral and topical acyclovir, carbamazepine, amoxicillin, and chlorhexidine digluconate. Teeth 27 and 28 were extracted due to hopeless prognosis. Fragments of necrotic alveolar bone were removed.
Kamala G.P. (2006) [14]	Case report	To present a case report of trigeminal *HZ* infection affecting the left maxillary and ophthalmic divisions of the fifth cranial nerve in an immunocompetent patient.	A 34-year-old male presented with a diagnosis of *HZ* in the ophthalmic and maxillary nerve, complicated by alveolar bone necrosis.	The patient was treated with aciclovir, erythromycin stearate, and ophthalmic medications. The patient responded favorably to the dental treatment and reported progressive improvement in vision in his left eye.
Florence E.C. (2013) [58]	Case report and literature review	To evaluate the safety and efficacy of azathioprine in *HIV*-infected individuals, focusing on potential adverse events, immune parameters, and hemoglobin levels.	Four females and three males with a mean age of 38. All patients were receiving highly active antiretroviral therapy (HAART). Thiopurine methyltransferase activity was tested prior to commencing treatment with azathioprine, and all individuals were assessed as tolerant.	The study concludes that azathioprine can be used in *HIV*-infected individuals with careful monitoring of immune parameters and hemoglobin levels. No serious opportunistic infections or malignancies were reported.
Rupinder K. (2016) [48]	Case report and literature review	To report a rare case of spontaneous tooth exfoliation associated with trigeminal *HZ* in a diabetic patient.	A 47-year-old male with crusty lesions of herpes infection over the left side of the face and high mobility in the left upper central incisor that subsequently exfoliated.	Spontaneous tooth exfoliation is a rare but significant complication of trigeminal *HZ*, particularly in patients with underlying diabetes mellitus.

## Data Availability

Not applicable.

## Abbreviations

HAART	Highly active antiretroviral therapy
*HSV*	*Herpes simplex virus*
HZI	Herpes Zoster infection
IOMA	Integrative omics-metabolic analysis
ONJ	Osteonecrosis of the jaw
PRISMA	Preferred Reporting Items for Systematic Reviews and Meta-Analyses
PROSPERO	The International Prospective Register of Systematic Reviews
RHS	Ramsey Hunt Syndrome
*VZV*	*Varicella zoster virus*
